# Effectiveness of a programme of exercise on physical function in survivors of critical illness following discharge from the ICU: study protocol for a randomised controlled trial (REVIVE)

**DOI:** 10.1186/1745-6215-15-146

**Published:** 2014-04-27

**Authors:** Brenda O'Neill, Kathryn McDowell, Judy Bradley, Bronagh Blackwood, Brian Mullan, Gavin Lavery, Ashley Agus, Sally Murphy, Evie Gardner, Daniel F McAuley

**Affiliations:** 1 Centre for Health and Rehabilitation Technologies (CHaRT), Institute of Nursing and Health Research, School of Health Sciences, University of Ulster, Newtownabbey BT37 0QB, UK; 2 Department of Respiratory Medicine, BHSCT, Belfast City Hospital site, Belfast BT9 7ER, UK; 3 Centre for Infection and Immunity, School of Medicine, Dentistry and Biomedical Sciences, Queen’s University of Belfast, Belfast BT9 7AE, UK; 4 Regional Intensive Care Unit, Royal Hospitals at BHSCT, Grosvenor Road, Belfast BT12 6BA, UK; 5 Northern Ireland Clinical Trials Unit (NICTU), The Royal Hospitals, Grosvenor Road, 1st Floor Elliot Dynes Building, Belfast BT12 6BA, UK

**Keywords:** Critical illness, Critical care, Intensive care unit, Rehabilitation, Exercise programme, Physical function, Health-related quality of life

## Abstract

**Background:**

Following discharge home from the ICU, patients often suffer from reduced physical function, exercise capacity, health-related quality of life and social functioning. There is usually no support to address these longer term problems, and there has been limited research carried out into interventions which could improve patient outcomes. The aim of this study is to investigate the effectiveness and cost-effectiveness of a 6-week programme of exercise on physical function in patients discharged from hospital following critical illness compared to standard care.

**Methods/Design:**

The study design is a multicentre prospective phase II, allocation-concealed, assessor-blinded, randomised controlled clinical trial. Participants randomised to the intervention group will complete three exercise sessions per week (two sessions of supervised exercise and one unsupervised session) for 6 weeks. Supervised sessions will take place in a hospital gymnasium or, if this is not possible, in the participants home and the unsupervised session will take place at home. Blinded outcome assessment will be conducted at baseline after hospital discharge, following the exercise intervention, and at 6 months following baseline assessment (or equivalent time points for the standard care group). The primary outcome measure is physical function as measured by the physical functioning subscale of the Short-Form-36 health survey following the exercise programme. Secondary outcomes are health-related quality of life, exercise capacity, anxiety and depression, self efficacy to exercise and healthcare resource use. In addition, semi-structured interviews will be conducted to explore participants’ perceptions of the exercise programme, and the feasibility (safety, practicality and acceptability) of providing the exercise programme will be assessed. A within-trial cost-utility analysis to assess the cost-effectiveness of the intervention compared to standard care will also be conducted.

**Discussion:**

If the exercise programme is found to be effective, this study will improve outcomes that are meaningful to patients and their families. It will inform the design of a future multicentre phase III clinical trial of exercise following recovery from critical illness. It will provide useful information which will help the development of services for patients after critical illness.

**Trial registration:**

ClinicalTrials.gov NCT01463579

## Background

Following discharge from the ICU, patients often suffer from reduced physical function, exercise capacity, health-related quality of life and social functioning which may continue for up to 5 years following discharge home from hospital [1-5]. In a systematic review of 53 studies (10 multicentre) reporting health-related quality of life outcomes at least 12 months after discharge from the ICU, critically ill patients had a lower health-related quality of life than an age- and gender-matched population [6]. The worst reductions in quality of life were seen in cases of severe acute respiratory distress syndrome, prolonged mechanical ventilation, severe trauma, and severe sepsis. There is usually no support to address these longer term problems specific to critical illness. Rehabilitation after critical illness is recognised as a prominent therapeutic objective for the future for this population in the recent National Institute for Health and Care Excellence Guidelines [7].

There is evidence to support the rehabilitation of critically ill patients within the ICU [8-10], but there is a paucity of literature to support rehabilitation following discharge from intensive care and particularly following discharge from hospital. It is also unclear what components should be included in post-hospital discharge rehabilitation. At the time of concept of this trial, studies evaluating post-hospital discharge rehabilitation following critical illness showed discordant results [11-14]. Jones and colleagues [11] conducted a randomised controlled trial comparing a rehabilitation manual, which included advice on psychological, psychosocial and physical problems and a self-directed exercise programme, to standard care. While there was significant improvement in the Short-Form-36 Health Survey (SF-36) physical function scores and reduced depression, the effects on outcomes such as functional ability or exercise capacity were not measured. McWilliams and colleagues [12] assessed the effect of a 6-week rehabilitation programme, which included education and supervised exercise classes as well as unsupervised home exercise sessions in a cohort group. It did not include blinded outcome assessment. Significant improvement in physical function using the six-minute walk test (6MWT) and the incremental shuttle walk test and the hospital anxiety and depression scores were found.

Two trials failed to demonstrate improved outcomes in the intervention group compared to standard care [13,14]. An 8-week home-based physical rehabilitation programme failed to show improvements in physical function (6MWT) and health-related quality of life (SF-36) compared to standard care [13]. Potential reasons reported for this lack of difference include a lack of compliance in the intervention group due to minimal supervision and inadequate training intensity. A nurse-led follow-up programme reported no difference in outcomes compared to standard care [14]. It is difficult to determine whether the results of this study were affected by the limited nature of the intervention which did not appear to include elements of rehabilitation that would target important physical and non-physical problems in this population.

In these studies [11-14], patients’ length of time in ICU and/or duration of mechanical ventilation were relatively short. It is debatable whether this patient cohort would have significant disability or reasonably represent the population that would benefit most from rehabilitation interventions. The present study, therefore, plans to address the gap in current knowledge by investigating the effectiveness of an exercise programme on physical function in patients discharged from hospital following critical illness compared to standard care. To overcome the limitations of previous studies, two out of three exercise sessions per week, for a 6-week period, will be supervised and we aim to recruit patients who have had a longer time on mechanical ventilation than in previous studies (greater than 96 hours) as they may be at higher risk of longer term sequelae after critical illness. In addition, we will assess the suitability of the programme, both from the patient’s perspective and a healthcare cost perspective.

### Aim

The aim of this study is to investigate the effectiveness and cost-effectiveness of a 6-week programme of exercise on physical function in patients discharged from hospital following critical illness compared to standard care.

### Objectives

The objectives are: (i) to investigate the effectiveness of a 6-week programme of exercise on physical function, health-related quality of life, exercise capacity, anxiety and depression and self efficacy to exercise in patients discharged home from hospital following critical illness compared to standard care; (ii) to determine the feasibility (safety, practicality and acceptability) of providing a 6-week programme of exercise for patients discharged from hospital following critical illness; (iii) to explore participants’ perceptions of the 6-week exercise programme; (iv) to examine medium-term (6-months) effects of the exercise programme; and (v) to evaluate the cost-effectiveness of the exercise programme.

## Methods/Design

**Figure 1:**
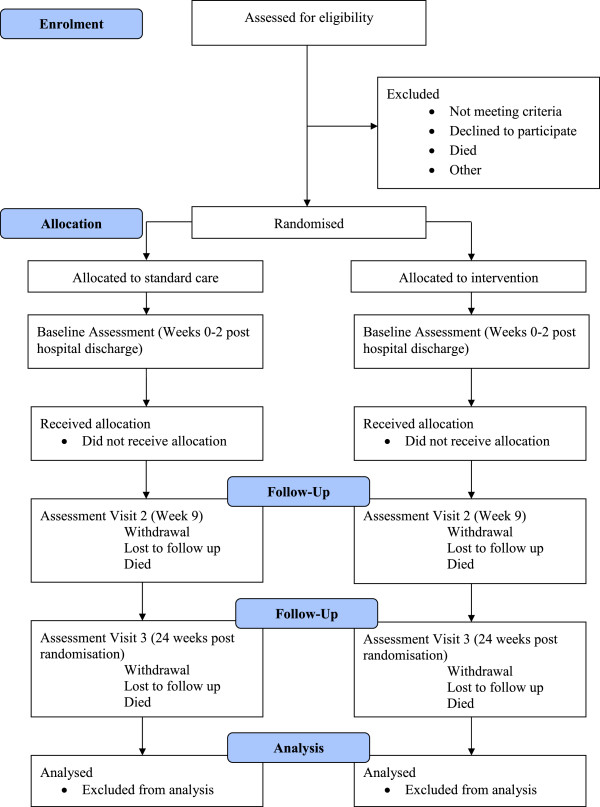
**Consolidated Standards of Reporting Trials (CONSORT) 2010 study flow diagram.** CONSORT flow diagram for effectiveness of a programme of exercise on physical function in survivors of critical illness following discharge from the ICU: study protocol for a randomised controlled trial.

The planned design of this multicentre prospective phase II, allocation-concealed, assessor-blinded, randomised controlled clinical trial adheres to the Standard Protocol Items Recommendations for Interventional Trials guidelines for clinical trial protocols [15] and the Consolidated Standards of Reporting Trials statement for reporting randomised controlled trials [16] (Figure 1).

### Study approvals

The study protocol has been approved by the Northern Ireland Research Ethics Committee (REC reference 11/NI/0115) and research governance approval has been granted by the five Health and Social Care Trusts in Northern Ireland. The study is registered with ClinicalTrials.gov registry (NCT01463579) [17]. It is sponsored by the University of Ulster and the Belfast Health and Social Care Trust, and has been adopted by the Northern Ireland Clinical Research Network (Critical Care and Respiratory Health). It is supported by the Northern Ireland Clinical Trials Unit (NICTU), a UK Clinical Research Collaboration registered clinical trials unit.

### Study population

To be eligible for recruitment into the study patients must be aged 18 years or older, have had an ICU admission requiring mechanical ventilation greater than 96 hours, planned discharge to home (self-care/carer), be willing and able to participate in exercise and deemed medically fit to take part in the intervention. Patients will be excluded if they meet any of the following criteria: declined consent or unable to give consent; inability to participate due to, for example, any neurological, spinal or skeletal dysfunction affecting ability to exercise; cognitive impairment affecting ability to consent, participate in the intervention or complete questionnaires; participation in another structured rehabilitation programme due to ongoing chronic disease; any medical contraindication to participation in an exercise programme.

### Recruitment

Patients in the ICUs will be prospectively screened. All eligible consecutive patients will be given written information about the study following discharge from critical care. They will be approached to enquire if they are interested and willing to take part in the study. A screening log of all non-recruited patients and the reason for exclusion will be maintained. If willing to participate, patients will be asked to provide written informed consent by a trained member of the research team prior to commencing the study. Participants will be randomised to the exercise programme or standard care. A randomisation schedule will be produced using permuted block randomisation technique, at a 1:1 ratio. Variable block sizes are used to ensure blinding. The randomisation schedule will be generated using nQuery Advisor by the clinical trials unit (NICTU). Allocations are done centrally by the NICTU (allocation concealment). The randomisation service will allocate a unique trial identification number to each participant in accordance with the computer-generated study randomisation schedule. In order to maintain confidentiality, all case report forms, study reports and all communication regarding the study will identify participants using the unique identification numbers. Participants allocated to the programme of exercise will commence as soon as possible.

### Sample size

There is limited data available in this specific research area in order to conduct a formal sample size calculation. Jones and colleagues [11] demonstrated an approximate 24% improvement in the physical function score of the SF-36 with a rehabilitation manual which included a self-directed exercise programme. With the limited data available we have used a more general method to calculate the sample size [18], which would result in 52 patients (26 per group) for a large effect size. On the basis of our previous experience and published data we have estimated a loss of 25% after randomisation [19]. Thus, this study will aim to recruit up to 68 patients (34 in each group), or until we achieve 52 (26 per group) completed datasets at 6 weeks over the proposed 36-month recruitment period. Participants will be withdrawn on request, if there are adverse effects arising from the exercise programme, or due to inability to perform baseline assessment.

### Recruitment strategies

Strategies to optimise recruitment to the trial will be implemented throughout the trial, and will include: adoption into a clinical trial network (Northern Ireland Clinical Research Network) to facilitate access to dedicated research staff trained in research methods and effective patient tracking procedures between Health and Social Care Trusts in Northern Ireland; weekly contact with recruitment sites to ensure timely resolution of patient eligibility queries; and early identification of reasons for patient decline and mechanisms to minimise patient barriers to recruitment.

### Standard care

Following ICU admission, standard care consists of discharge to a hospital ward to the care of the ward consultant, and the patients are no longer under the care of the critical care team. They are provided with appropriate medical and nursing care, and with referral to other disciplines as necessary. Once mobile and able to return home to a carer or another facility they are discharged from hospital. Participants randomised to the standard care group will receive no additional support to address potential problems specific to critical illness from the study.

### Intervention (exercise programme)

Participants randomised to the intervention group will complete three exercise sessions per week (two sessions of supervised exercise and one unsupervised session) for 6 weeks. Supervised sessions will take place in a hospital gymnasium or, if this is not possible, in the participants home and the unsupervised session will take place at home. Participants will be informed that they can invite a relative or carer to attend the sessions. The selection of a supervised mostly outpatient-based structured exercise programme is important in this study to promote participant confidence in their ability to exercise in a group setting with people with similar problems and experiences of critical illness, and at the same time facilitate an appropriate level of monitoring and safety [20].

A circuit of exercises will be used to provide interval training for up to 60 minutes and this will include rest periods and an aerobic component [19-23]. The programme of exercise will be delivered by trained physiotherapists. It will consist of a warm-up period, a circuit of 10 arm, leg and whole body conditioning and strengthening exercises, followed by an additional period of aerobic exercise (for example, walking, cycle ergometry or treadmill walking) to maintain moderate breathlessness, and finally by a cool-down period and relaxation. Although the programme is standardised it will be tailored to the capability of each individual participant and exercises will be progressed to maintain a level of moderate breathlessness [24]. Strengthening exercises will be included, using higher repetitions and sets and an increase in weight for progression. The aerobic exercise can be based, for example, on the patient’s heart rate and/or the results of the incremental shuttle walk test measured at baseline assessment. All participants will be asked to rest for 10 minutes following cessation of the exercise programme.

Participants will be provided with a written and illustrated exercise manual which facilitates completion of their exercise programme during supervised and unsupervised sessions. The exercise manual has been developed and reviewed by patient representatives, physiotherapy clinicians and the research team. It contains information on the importance of exercising after critical illness, safety advice relating to exercise and information on ‘how you should feel when you exercise’ which details the Borg breathlessness scale [24], as well as a written plan to support completion of the exercises when the participant is at home. Adherence with the sessions will be recorded on a weekly basis using a diary. This approach has been successfully used in a previous exercise-based trial [19]. At the end of the 6-week programme, participants will receive a short consultation to set goals relating to continuing exercise at home and advice to follow their set goals and use a diary to support continued self-directed exercise and physical activity.

### Risks to participants from the intervention

The risk relating to the intervention is minimised as patients who are not suitable to undertake exercise are excluded, and participants will not be asked to undertake exercise beyond their physical ability. The exercise sessions will be delivered by trained physiotherapists who will work closely with the ICU clinical team, and they will have frequent contact and access to the ICU team for any queries that may arise. The records and baseline assessment of all participants included in the study will be reviewed by an ICU physician.

### Researcher safety

On occasions when home visits are required the researchers will follow the research group safety protocol for home visits. When scheduling the home visit the researcher will conduct a preliminary risk assessment. Prior to the home visit they will complete an offsite visit itinerary form, including a nominated contact person’s details and a call schedule. The contact person will be another member of the research team available during the visit.

### Data collection

Blinded outcome assessment will be conducted at baseline within two weeks of hospital discharge or when the patient is deemed medically stable (or in exceptional circumstances this will take place immediately prior to hospital discharge), following the exercise intervention, and at 6 months (24 weeks) following baseline assessment (or equivalent time points for the standard care group). These will be conducted by trained independent assessors blinded to group allocation. Informed consent will be obtained from each participant. Following consent and randomisation, the following will be recorded: demographics, ICU diagnosis and any co-morbidity and illness severity using acute physiology and chronic health evaluation II score.

### Outcome measures

The primary outcome measure is physical function as measured by the physical functioning subscale of the SF-36 following the exercise programme. This has been shown to be an acceptable, reliable and valid tool following critical illness [25].

Several secondary outcomes will be evaluated to determine whether an exercise programme can improve these important clinical outcomes. These outcome measures will assess physical function, health-related quality of life, exercise capacity, anxiety and depression and self-efficacy to exercise in these participants. Healthcare resource use will also be evaluated [25-35] (Table 1).

**Table 1:** Secondary outcome measures

**Outcome measure**	**Instrument**
Physical function	Rivermead Mobility Index [26]
Hand function (strength and dexterity)	Dynamometry [27]
	Nine Hole Peg Test [28]
Exercise capacity	Incremental Shuttle Walk Test [29]
Health-related quality of life	Other subscales of the Short-Form-36 Health Survey (role limitations due to physical health, bodily pain, general health perceptions, vitality, social functioning, role limitations due to emotional problems, and mental health) [25]
	Functional Limitations Profile [30]
	EuroQol 5 dimension questionnaire 5 level version [31]
Breathlessness	Medical Research Council Dyspnoea Scale [32]
Anxiety and depression	Hospital Anxiety and Depression Scale [33]
Readiness to exercise	Readiness to Change Questionnaire [34]
Self efficacy to exercise	Chronic Disease Self Efficacy Scale [35]
Healthcare resource use	Resource use questionnaire

Feasibility (safety, practicality and acceptability) of providing the exercise programme will also be assessed by the occurrence of adverse events, and recording information relating to recruitment and retention, and any difficulties with attendance and transport. Information regarding ease of execution of the exercise programme and the outcome measures will also be recorded.

In addition, we will conduct semi-structured interviews to explore participant’s perceptions of the exercise programme. On completion of the exercise programme semi-structured interviews will be used to explore participant satisfaction, views about continuing exercise and suggestions for improving the exercise programme. A list of clear, open-ended questions will be prepared by the research team [36]. The interviews will be carried out by a trained researcher not involved in the delivery of the intervention or blinded outcome assessment. All interviews will be audio taped, transcribed and analysed. Finally, the results from this qualitative component, along with the results from the outcome measures from the trial, will provide greater insight into both the process and effects of the intervention.

### Statistical methods/analyses

For continuously distributed outcomes, differences between groups will be tested using independent samples *t*-tests, analysis of variance and analysis of covariance with transformations of variables to normality if appropriate, or non-parametric equivalents. Chi-square tests (or Fisher’s Exact tests) will be used for categorical variables. Efficacy of intervention will be analysed on an intention-to-treat basis. A *P* value ≤0.05 will be considered significant. A single final analysis is planned at the end of the trial. Baseline variables will be recorded to demonstrate that the groups are comparable; if this is not demonstrated, *post-hoc* adjustments to correct for differences will be applied. Every effort will be made to collect outcomes from patients, even those who drop out of the exercise programme. Every effort will be made to minimise missing baseline and outcome data in this trial. The level and pattern of the missing data in the baseline variables and outcomes will be established by forming appropriate tables and the likely causes of any missingness will be investigated. This information will be used to determine whether the level and type of missing data has the potential to introduce bias into the analysis results for the proposed statistical methods, or substantially reduce the precision of estimates related to treatment effects. If necessary, these issues will be dealt with using multiple imputation or Bayesian methods for missing data as appropriate. The power calculations and methodology for data analysis have been reviewed and confirmed by the study statistician. Until data analysis is completed the study statistician will be unaware of whether the participant received the intervention or standard care.

### Health economic evaluation

A within-trial cost-utility analysis will be undertaken to assess the cost-effectiveness of the intervention compared with standard care. The perspective will be that of the National Health Service and Personal Social Service as recommended by the National Institute for Health and Care Excellence [37]. The outcome of the cost-utility analysis will be the cost per quality-adjusted life year (QALY) gained. QALYs will be calculated using utilities generated from responses on the EuroQol 5 dimension questionnaire 5 level version administered over the study period. Healthcare resource use over the study period will be collected using a questionnaire administered at 6 months post-randomisation. Overheads, capital and patient training costs will be determined based on an average exercise class. Intervention costs will also be captured and will include the costs associated with staff training and delivery of the intervention. Unit costs will be applied from published national sources such as the National Health Service Reference Costs [38], British National Formulary, and the Unit Costs of Health and Social Care [39]. An incremental cost effectiveness ratio will be calculated to estimate the cost per QALY gained. Patient level cost and QALY data will be bootstrapped and a cost-effectiveness acceptability curve will be derived to show the probability of the intervention being cost-effective compared to usual care at various thresholds of willingness-to-pay for an additional QALY. Sensitivity analysis will be performed to explore the impact on cost-effectiveness of variations in key parameters. Costs which might be borne by the patient will also be explored. Given the study timeframe, discounting will not be necessary.

### Study quality and monitoring procedures

Rigorous study conduct and monitoring procedures will be put in place to ensure the research quality and data is of a high standard and to ensure any problems are identified and managed.

A Data Monitoring and Ethics Committee (DMEC) has been appointed to the study. The DMEC is independent of the study organisers. They will independently review cumulative safety data to determine whether the trial should continue as originally designed, should be changed, or should be terminated based on these data. Following each DMEC meeting, the DMEC Chair will provide written information of any recommendations related to continuing, changing, or terminating the trial to the principal investigator. The recommendations will be shared with the sponsor and the Research Ethics Committee.

An electronic case report form has been developed for the study by the NICTU, including validation checks to minimise missing data and errors. Outcome measures and other key data will also be monitored by an independent data monitor for any errors, missing data or inconsistencies.

Formal monitoring of study procedures at each site will take place at least once per year over the course of the study and as required thereafter. Regular review of training needs and training updates as required will be completed with the researchers carrying out the study procedures.

The study is sponsored by the University of Ulster and the Belfast Health and Social Care Trust whose role includes ensuring that good practice arrangements are maintained for the duration of the study in relation to the conduct of the study, monitoring and reporting. Participant safety and well-being will be protected by implementing standard operating procedures as set out in the Research Governance Framework, including the reporting and recording of adverse events. The study participants are covered by indemnity for negligent harm through the standard sponsor indemnity arrangements.

The usual regulations will be followed for communicating important protocol modifications to the sponsors, Research Ethics Committee and governing sites as appropriate and the trial registries will be updated accordingly.

In order to support translation of our results to clinical practice we will disseminate the findings of this study to relevant health professionals through conference presentation and publication in peer-reviewed journals. Authorship eligibility guidelines will be followed. Furthermore, we will provide information for the public via appropriate forums. Trial participants will be informed that they will be sent a summary of the results on request.

## Discussion

Survivors of critical illness have persistent reduced physical function and health-related quality of life, and ongoing healthcare utilisation. There is also significant negative impact in terms of economic, social, physical and psychological factors on those who care for survivors of critical illness following their discharge home [40]. The desired outcome following critical illness is the return to physical function and quality of life levels similar to those experienced before the critical illness.

This multicentre prospective phase II, allocation-concealed, assessor-blinded, randomised controlled clinical trial will investigate the effectiveness of a programme of exercise on physical function in patients discharged from hospital following critical illness compared to standard care. There is limited research exploring the effects of rehabilitation after discharge from hospital following critical illness and so this study will advance the knowledge and research in this area. Additionally it includes some design elements in the inclusion criteria, methodology, intervention and outcome assessments which are not included in other studies and will provide further added value. We aim to recruit patients who have had a longer time on mechanical ventilation in ICU than in previous studies (greater than 96 hours) so that the population are at higher risk of longer term sequelae after critical illness and therefore more likely to represent the population that would benefit most from rehabilitation interventions. By following the principles for the prescription of exercise outlined by the American College of Sports Medicine and professional bodies [20-23], the proposed intervention seeks to be as effective as possible in order to improve outcomes for these patients. Two out of three exercise sessions per week, for a 6-week period, will be supervised. Although standardised, the programme of exercise will be tailored and progressed to the capability of each individual. The structured and self-directed exercise manual will be included to support the intervention [7], and a consultation at the final exercise session relating to continuing exercise.

If effective, this study will improve outcomes that are meaningful to patients and their families, and inform the design of a future multicentre phase III clinical trial of exercise following recovery from critical illness. It will provide useful information which will help the development of services for patients after critical illness.

## Trial status

Recruitment is currently active in five recruitment sites [17] and the first patient was randomised in January 2012. It is anticipated that study accrual will be completed by December 2014.

## Abbreviations

6MWT: six-minute walk test; DMEC: Data Monitoring and Ethics Committee; NICTU: Northern Ireland Clinical Trials Unit; QALY: quality-adjusted life year; SF-36: Short-Form-36 Health Survey.

## Competing interests

All authors declare that they have no competing interests.

## Authors’ contributions

DFMcA, SM, GL and BO’N conceived the study and, in collaboration with KMcD, BB, JB, BM and AA, wrote and refined the trial protocol and developed it for funding. EG verified the sample size for the study and proposed data analysis. All authors have made a substantial contribution to the protocol development, design of the study and initiation and set up of the study. All authors have approved the final manuscript to be published.
